# Next-generation development and application of codon model in evolution

**DOI:** 10.3389/fgene.2023.1091575

**Published:** 2023-01-27

**Authors:** Manoj Kumar Gupta, Ramakrishna Vadde

**Affiliations:** Department of Biotechnology & Bioinformatics, Yogi Vemana University, Kadapa, Andhra Pradesh, India

**Keywords:** codon substitution models, mechanistic model, empirical model, semi-emperical models, evolution, phylogenetic reconstruction

## Abstract

To date, numerous nucleotide, amino acid, and codon substitution models have been developed to estimate the evolutionary history of any sequence/organism in a more comprehensive way. Out of these three, the codon substitution model is the most powerful. These models have been utilized extensively to detect selective pressure on a protein, codon usage bias, ancestral reconstruction and phylogenetic reconstruction. However, due to more computational demanding, in comparison to nucleotide and amino acid substitution models, only a few studies have employed the codon substitution model to understand the heterogeneity of the evolutionary process in a genome-scale analysis. Hence, there is always a question of how to develop more robust but less computationally demanding codon substitution models to get more accurate results. In this review article, the authors attempted to understand the basis of the development of different types of codon-substitution models and how this information can be utilized to develop more robust but less computationally demanding codon substitution models. The codon substitution model enables to detect selection regime under which any gene or gene region is evolving, codon usage bias in any organism or tissue-specific region and phylogenetic relationship between different lineages more accurately than nucleotide and amino acid substitution models. Thus, in the near future, these codon models can be utilized in the field of conservation, breeding and medicine.

## Introduction

The continuous growth of DNA and protein data has provided an opportunity to infer their function and their evolutionary history of any sequence/organisms in a more comprehensive way (Anisimova and Liberles, 2007; Miyazawa, 2011a; Dufresne and Jeffery, 2011; Gupta and Vadde, 2019a; Gupta et al., 2019; Gouda et al., 2020; Gupta et al., 2021a; Gupta et al., 2021b; Gupta et al., 2021c; Gupta et al., 2021d; Gupta et al., 2021e; Chu et al., 2021). Population genetics and phylogenetics are two of the most important subfields for inferring the evolutionary history of any sequences/organisms (Haubold, 2014). While phylogeny approaches infer the evolution of species and higher taxonomic orders, population genetics approaches are generally used for understanding the evolution of the groups below the species level (Haubold, 2014). It is pertinent to note that there is only one diagram, a phylogeny, that appears in Darwin’s seminal work, “The Origin of Species.” This, in turn, indicates that phylogenies are the core metaphor of evolutionary biology, and efforts to create them is as old as the evolutionary science field itself. Phylogenies relation are inferred by comparing homologous characteristics that differ (Haubold, 2014). A phylogenetic tree, often known as an evolutionary tree, is a diagrammatic depiction of the evolutionary relationship between different species (Gupta et al., 2021c). All phylogenetic tree analysis is based on certain implicit/explicit hypothetical models that make the complex biological process into simpler form (Gatto et al., 2007; Zaheri et al., 2014). However, the validity of certain models could be plausibly challenged while analyzing real data. For instance, the JC69 model hypothesizes that the rate of nucleotide substitution is the same for all pairs of the four nucleotides, namely, guanine (G), cytosine (C), adenine (A), and thymine (T) (Jukes and Cantor, 1969). However, in reality, there are numerous mutations, and some mutations are less tolerated in comparison to others (Liò and Goldman, 1998; Choudhuri, 2014). Nevertheless, all models share a common assumption, i.e., the Markov property (Liò and Goldman, 1998). In probability theory, any stochastic process has the Markov property if the probability distribution of future states of the process is dependent only on the present state (Gudivada et al., 2015). Markov property has been widely utilized in population genetics research to understand the change in gene frequencies in small populations affected *via* genetic drift (Watterson, 1996).

Based on sequence type, all these substitution models can be broadly classified as nucleotides, amino acid, and codon substitution models (Arenas, 2015b). The parameter space dimension for these models varies from 4 × 4 nucleotide substitution model to 20 × 20 amino acid substitution models and, finally, to 61 × 61 codon substitution models (where stop codons are generally omitted). Because of only four states and small physiochemical differences between base properties, nucleotide substitution models are easily modeled *via* Markov models (Yang, 2006). However, as natural selection functions mostly at the protein level, estimating evolutionary history based on nucleotide substitution models can sometimes be misleading (Shapiro et al., 2006; Seo and Kishino, 2008). Both amino acid or codon substitution models consider protein-coding sequences and thus, an evolutionary distance estimated *via* them is more accurate than the evolutionary distance estimated through nucleotide substitution models (Anisimova and Kosiol, 2009). Nevertheless, due to the complex physiochemical relationship between amino acids, it is often difficult to predict the substitution rate between amino acids in a small set of the original dataset. Hence, the substitution rate in amino acid substitution models is generally estimated from pre-defined empirical data sets (Anisimova and Kosiol, 2009), which in turn may predict evolutionary history less accurately.

The codon substitution models are especially interesting for protein-coding genes because they consider both mutational propensities at the nucleotide level and selective pressure on amino acid substitutes as well as genetic code for estimating evolutionary distance (Sullivan and Joyce, 2005). Additionally, amino acid substitution models can estimate only purifying selection acting on each site of sequence, whereas codon substitution models can estimate both purifying as well as positive Darwinian selection (Doron-Faigenboim and Pupko, 2007). Even for highly divergent species, phylogenetic trees constructed *via* codon models were reported to be more accurate than the phylogenetic tree constructed through the amino acid substitution model (Zaheri et al., 2014). Though these models were neglected initially, tracing phylogenetic relationships between populations and traits/diseases *via* codon substitution models is increasing nowadays in evolutionary medicine research (Grunspan et al., 2017). Thus, the codon substitution model is more powerful than nucleotide (Kimura, 1980; Hasegawa et al., 1985; Tamura and Nei, 1993) and amino acid (Dayhoff et al., 1978; Jones et al., 1992; Adachi and Hasegawa, 1996) substitution models. However, as codon substitution models are computationally more demanding, their usage is minimal. Hence, it needs to develop more robust but less computationally demanding codon substitution models for reconstructing evolutionary history from sequence data. In this review article, authors made an attempt to understand the basis of the development of different types of codon-substitution models and how this information can be utilized to develop more robust and less computationally demanding codon substitution models for more accurate phylogeny as well as understand the evolutionary history of any sequences or organisms. In the near future, these models can be applied in the field of conservation, breeding and medicine.

## Basis of development of substitution models

Evolution is generally considered a stochastic process by which the DNA segment can be either inserted or deleted, or duplicated, or recombination may take place (Cannarozzi and Schneider, 2012). The most frequent events during evolution are point mutation, which may have either no effect or a small effect or change protein function completely. If this point mutation becomes fixed, either due to genetic drift/positive selection, it is called substitution (Cannarozzi and Schneider, 2012). The probability by which a new base gets fixed in a population is dependent on the accompanying modification in the species’ fitness (Cannarozzi and Schneider, 2012). To date, numerous hypothetical substitution models have been proposed to understand the mechanism associated with substitutions in either nucleotide or amino acid sequences (Cannarozzi and Schneider, 2012). Though some of these models are more complex than others, all substitution models share a common assumption, i.e., the Markov property (Liò and Goldman, 1998). Utilizing the Markov property, these substitution models (Markov model) estimate the probabilities for possible temporal or sequential DNA or protein sequences in any individual or species. They also enable us to detect a preference of any sequences towards the GC or AT content (Jukes and Cantor, 1969).

Each Markov model has some parameters, which, in evolutionary biology, either represent the substitution rate or from which the substitution rate can be derived (Cannarozzi and Schneider, 2012). These parameters and hypotheses related to these parameters are often estimated *via* Maximum Likelihood (ML) approaches (Yang, 2006). ML detects optimum parameters associated with the occurrence of data (for instance, a group of nucleotide sequences) under a given phylogenetic tree and specific evolutionary model (Fisher, 1925; Edwards, 1972). DART (DNA, Amino acid, and RNA Tests) (Holmes and Rubin, 2002) and PAML (“Phylogenetic Analysis by Maximum Likelihood”) (Yang, 2007) are two of the most widely used software for estimating phylogenetic ML. Apart from the phylogenetic tree, the ML value can also be utilized for answering several other biological questions. For instance, the identification of the most extreme transition/transversion mutational biases in a set of aligned sequences and sites that are evolving under the greatest selective constraints (Fisher, 1925; Edwards, 1972; Felsenstein and Felenstein, 2004).

These days, codon substitution models are also developed in a Bayesian framework. Apart from the likelihood function, Bayesian inference also describes a prior likelihood distribution on the model parameter (Benner, 2012). Thus, the main objective of the Bayesian inference is to compute the posterior distribution (which is proportional to the likelihood multiplied by the prior), acquire random samples from this posterior density by means of Monte Carlo (MC) methods, and estimate mean and other numerous quantities on the basis of the sample obtained (Benner, 2012). Theoretically, any ML model can be converted into a Bayesian model just by adding a prior distribution. The MC methods employed in Bayesian inference are often very powerful in exploring entirely novel codon substitution models, which is not possible by using classical numerical optimization approaches. For instance, the likelihood function, which is to be numerically assessed point-wise, as well as optimized with regard to its parameters, is itself a complicated integral over random variables (Benner, 2012). This integral is present analytically available only for the simplest models. On the contrary, when complicated models are considered, analyticity gets depleted, and the classical numerical optimization approaches employed in recent ML fail. In contrast, MC sampling approaches permit numerous algorithmic tricks, for instance, data augmentation as well as parameter expansion, which in turn improvise the requirement for obvious analytical integration over incomplete observations or over auxiliary variables. Nevertheless, MC methods are very demanding in the context of both code development as well as computational cost (Benner, 2012). Thus, the number of Bayesian MCMC methods developed to date is very few and still, we have to wait for the joint availability of huge amounts of inter-specific sequence data as well as more powerful computational facilities for understanding their potential in a more comprehensive way (Benner, 2012). Thus, the Markov property or Bayesian framework is the basis for the development of almost all substitution models developed to-date.

## Substitution models

Based on sequence type, substitution models can be broadly classified as (a) nucleotide, (b) amino acid, and (c) codon substitution models.

### Nucleotide substitution model

JC69 model is the simplest model of nucleotide substitution (Liò and Goldman, 1998). It is based on two simple assumptions. The first assumption is that each residue of DNA is equally likely to change to any of the other three nucleotide bases. The second assumption is that all four bases have the same frequency. Hence, the rate of transition is equal to the rate of transversions (Pevsner, 2015). Because of its simple assumptions, the JC69 model is unlikely to be applicable in most of the data sets and works reasonably only in closely related sequences. Though it can be utilized in distantly related sequences, the correction made can sometimes be too significant to be reliable. As this model involves a single parameter, α , for both the rate of substitution for each nucleotide (3 α per unit time) and the rate of substitution in each of the three possible directions of change ( $\left.\alpha \right)$ , it is called a one-parameter model. Kimura 2 Parameter (K80) is an extension of the JC69 model (Jukes and Cantor, 1969). As in real data, transversions generally occur at lower rates than transitions; (Kimura, 1980) proposed a model, which assumes that the transitions rate is different from the transversions rate. However, like the JC69 model, Kimura also assumes that all four bases have the same frequency.

Later several more robust nucleotide substitution models, like, F81 (Felsenstein, 1981), HKY85 (Hasegawa et al., 1985) and TN93 (Tamura and Nei, 1993) models, were developed. The F81 model was developed by American scientist (Felsenstein, 1981). Unlike Jukes & Cantor or Kimura-2 parameters, the F81 model assumes that the base frequency of all bases is different. However, like Jukes & Cantor, the F81 model assumes that the base substitution occurs with equal probability (Felsenstein, 1981). Hasegawa-Kishino-Yano 85 (HKY85) model assumes unequal base frequencies as well as different substitution rates between transversions and transitions (Hasegawa et al., 1985). Tamura and Nei’s 1993 (TN93) model assumes unequal base frequencies, but all transversions are assumed to take place at an equal rate, but the transition rate between purine differs from that of pyrimidine (Tamura and Nei, 1993).

For the first time in 1986, Simon Tavaré described a general independent, finite-sites, neutral, and time-reversible model called the general time-reversible **(**GTR**)** model or the general reversible **(**REV**)** model, which assumes different substitution rates for each pair of nucleotide and unequal base frequencies (Tavaré, 1986). Additionally, the rate of variation across sites (+G) (Yang, 1994a) and/or a proportion of invariable sites (+I) (Shoemaker and Fitch, 1989) can also be included in any model. Recently several other DNA substitution models comprising of non-stationary (nucleotide composition can change over time) and non-reversible (asymmetric) matrices (Boussau and Gouy, 2006; Jayaswal et al., 2011) or even involving neighbor interactions (Lunter and Hein, 2004) were developed for inferring phylogenetic trees more accurately.

### Amino acid substitution model

The two commonly used amino acid substitution matrices are the PAM matrices (Dayhoff et al., 1978) and the Blocks amino acid substitution matrices (BLOSUM) matrices. Margaret Dayhoff and the team aligned closely related protein sequences of seventy-one groups (Dayhoff et al., 1978). As all the sequences were closely related homologs, mutation detected in them were less likely to change the function of the protein, and hence the matrix designed was named PAM, which is an abbreviated form of Percent Accepted Mutations, where “accepted” designate the mutation favored *via* natural selection in the sequence (Xiong, 2006). The PAM matrices were generated on the basis of the evolutionary divergence amongst sequences of the same group. For instance, one PAM unit is described as 1% of amino acids have been modified (Xiong, 2006) and PAM60 is generated when the PAM1 matrix is multiplied by itself sixty times. Thus, PAM with a lower serial number is suitable for aligning closely related sequences, and PAM with a higher serial number is suitable for divergent sequences (Xiong, 2006). Later, Jones and the team utilized PAM matrices and developed a more advanced replacement matrix, namely the JTT model based on a large sequences dataset. After constructing a phylogenetic tree of each protein family, this method identified sequence pairs that are >85% identical and nearest-neighbors. Further, it also calculated the evolutionary distance among them. This pair of sequences were subsequently removed for avoiding recounting modifications on any given branch of a phylogeny. Likewise, this complete process was repeated for all such pairs of sequences in all protein families until the JTT matrix was finally developed (Jones et al., 1992).

BLOSUM is constructed on the basis of >2,000 conserved amino acid arrangements representing 500 groups of diverse protein sequences (Henikoff and Henikoff, 1992). Unlike PAM matrices, the BLOSUM matrices indicate the actual identity percentage amongst sequences selected for constructing the matrices (Henikoff and Henikoff, 1992). For instance, BLOSUM52 represents that sequences nominated for generating matrix share an average identity value of 52%. Hence, a higher BLOSUM number represents less divergent sequences. As PAM matrices, except PAM1, are generated from an evolutionary model and BLOSUM matrices are generated from direct observations, PAM matrices have more evolutionary meaning as compared to the BLOSUM matrices. Hence, PAM matrices are generally utilized for reconstructing phylogenetic trees. Nevertheless, due to the mathematical extrapolation technique utilized, the PAM matrices are less realistic for divergent sequences. The BLOSUM matrices are generated from local sequence alignments of conserved sequence blocks, while the PAM1 matrix is generated based on the global alignment of full-length sequences comprising both variable and conserved regions. Hence, BLOSUM matrices are more advantageous during database searching as well as finding conserved domains within proteins (Henikoff and Henikoff, 1992).

Later, (Adachi and Hasegawa, 1996), Yanga & team (Yang et al., 1998), and Adachi & team (Adachi et al., 2000) utilized the Maximum Likelihood method for developing vertebrate mitochondrial, mammalian mitochondrial (mtMAM), and chloroplast sequences (cpREV) specific amino acid replacement models, respectively. As Adachi & team, Yanga & team, and Adachi & team utilized only 20, 23 and 10 sequences, respectively, for constructing an amino acid replacement model, the accuracy of their matrices is always under question (Whelan and Goldman, 2001). Later Whelan and Goldman combined the best attributes of both Maximum Likelihood (ML) and counting methods for developing a more powerful amino acid replacement model from an extensive database of different globular protein families (Whelan and Goldman, 2001). Recently, Le and the team have also developed a more robust amino acid substitution model for metazoan mitochondrial (mtMet), vertebrate mitochondrial (mtVer) and invertebrate mitochondrial (mtInv) (Le et al., 2017). Amino acid substitution models have also been developed for Influenza virus (FLU) (Dang et al., 2010), HIV between-patient matrix HIV-Bm (HIVb) (Nickle et al., 2007), HIV within-patient matrix HIV-Wm (HIVw) (Nickle et al., 2007), arthropod mitochondrial (mtART) (Abascal et al., 2007), retrovirus (rtREV) (Dimmic et al., 2002) and general ‘Variable Time’ matrix (VT) (Müller and Vingron, 2000).

### Codon substitution models

A codon is a continuous three DNA/RNA bases sequences, which encodes a specific amino acid or stop signal during protein synthesis. As there are four different nucleotides, there are only 64 possible codons. Out of these 64, only 61 code for specific amino acids, while rest three codes act as a stop codon. Since there are only 20 amino acids, more than one codon encodes one amino acid. This degeneracy property of genetic code enables us to distinguish between synonymous (do not alter encoded amino acid) and non-synonymous (alter encoded amino acid) substitution at the nucleotide level (Yang et al., 2000). Codon models are generally utilized to estimate evolutionary pressures on proteins across divergent lineages *via* comparing the ratio of substitution rates at non-synonymous (dN) and synonymous sites (dS) in the protein-coding regions (ω = dN/dS). Employing synonymous polymorphisms as a proxy of neutral diversity, one can estimate if non-synonymous polymorphisms are hindered or favored by natural selection. In the neutral evolving genes, the fixation rate of non-synonymous and synonymous mutation will be the same (ω = 1). During negative (purifying) selection, the non-synonymous mutation is not favored by natural selection and thus is eliminated, causing the fixation rate of non-synonymous mutation to be lower than the synonymous rate (ω< 1). During positive (adaptive) selection, the non-synonymous mutation is favored *via* Darwinian selection, thereby causing the fixation rate of non-synonymous mutation to be higher than the synonymous rate (ω> 1) (Gupta and Vadde, 2020).

A study reported that ancient proteins are under strong purifying selection, while newly developed proteins are under positive selection (Vishnoi et al., 2010). As newly developed young genes perform either highly specialized (if generated *de novo* or *via* horizontal transfer) or redundancy (if generated *via* duplication) functions, they are more at risk of either losing their function or gaining novel functions in succeeding lineages (Domazet-Loso and Tautz, 2003; Daubin and Ochman, 2004; Wolf et al., 2009; Vishnoi et al., 2010). Though initially, young genes experience a large number of adaptive mutations, the substitution of some of the mutations will slowly optimize the function of the gene in due course of time and hence the supply of new adaptive mutations will also reduce; hence, ω value of a young gene will decline over time (Vishnoi et al., 2010; Moutinho et al., 2022). On the contrary, functions of old genes, like diabetic genes, are highly optimized and they are likely to have already exhausted all beneficial mutations by recent times and; thus, they are expected to evolve under negative selection and fix only neutral and/or nearly neutral mutations (Vishnoi et al., 2010).

Though ω was originally designed for detecting selective pressure acting on a protein across divergent lineage, ω can also be utilized for detecting selective pressure acting on a protein in a single population (Kryazhimskiy and Plotkin, 2008). However, selective pressure estimated *via* ω on sequences sampled from a single population differs from that of the divergent lineages. For instance, though ω< 1 is a clear signature of negative selection across divergent lineages, weak negative or strong positive selection between population samples is also expected to produce ω< 1 (Roumagnac et al., 2006; Holt et al., 2008; Kryazhimskiy and Plotkin, 2008). Strong positive selection in a population will generate speedy sweeps at selected sites (but not at neutral sites, which are presumed to be independent). Thus, two individuals from the same population under strong positive selection are likely to contain identical alleles at each selected site, generating ω< 1 (Kryazhimskiy and Plotkin, 2008).

## Approaches to estimate selective pressure on the coding region of a gene

To date, numerous methods have been developed for estimating selective pressure on the coding region of a gene. Most models consider numerous factors like codon biases and variation amongst sites to estimate selective pressure more accurately. Initial models were designed to estimate global ω for the entire sequence or for subsequences utilizing a sliding window approach. However, in reality, ω varies amongst each amino acid site in sequence data or amongst each branch in a phylogeny. Recently more advanced approaches were developed to predict ω per amino acid site (Yang, 2002; Suzuki, 2004b), which enable the identification of single sites under positive selection in spite of low global ω value for the entire protein. All these models can be broadly classified as mechanistic, empirical and semi-empirical codon substitution models.

### Mechanistic codon substitution models

Mechanistic codon models detect selective pressure on the coding region of a gene utilizing a finite set of parameters, for instance, synonymous/non-synonymous rate ratio, transversion/transition rate ratio, and codon frequencies at equilibrium. As the mechanistic codon model utilizes a finite set of parameters, it is also known as the parametric codon substitution model**.** Mechanistic codon models focus mainly on silent-transversion, silent-transition, replacement-transversion rates, and replacement-transitions amongst sense codons and codon frequencies. Considering all parameters in a single codon model will be computationally more demanding. Thus to avoid this problem, several mechanistic codon models have been developed to date. Each mechanistic codon substitution models have distinctive parameters that differentiate the substitution rate at the nucleotide level and selective pressure at the protein level. Thus, each mechanistic model has the capacity to estimate selective forces acting on any protein in their unique way (Whelan et al., 2001; Delport et al., 2009). If selective pressure at the protein level is not considered, codon models will be equivalent to nucleotide substitution models. If the substitution rate at nucleotide is not considered, the codon model will be equivalent to amino acid substitution models (Miyazawa, 2011a). Several studies utilizing a large set of protein-coding sequences reported that codon substitution models are statistically more powerful than nucleotide and amino acid models (Seo and Kishino, 2009; Miyazawa, 2011b). However, the codon model having a larger substitution rate was reported to be equivalent to the amino acid substitution model (Seo and Kishino, 2008).

The first two mechanistic codon substitution models (Goldman and Yang, 1994; Muse and Gaut, 1994) were capable of estimating only the global ω of the coding region of a gene. These two models considered transition/transversion ratio and codon frequencies for estimating ω (Goldman and Yang, 1994; Muse and Gaut, 1994). Besides, Goldman and Yang (Goldman and Yang, 1994) also considered replacement probabilities amongst amino acids on the basis of the Grantham physicochemical distance matrix (Grantham, 1974). Later Nielsen and Yang (Nielsen and Yang, 1998) & Yang and the team (Yang et al., 2000) developed more robust mechanistic Bayesian models individually. It is pertinent to note that in the models developed by Goldman and Yang (Goldman and Yang, 1994) and Nielsen and Yang (Nielsen and Yang, 1998), the rate of substitution is proportional to the frequency of the target codon (which is not very mechanistic), and later many models employes these models to “explain” the stationary distribution in codons, whereas in the model developed by Muse and Gaut (Muse and Gaut, 1994), it is proportional to the target nucleotide, which is much more mechanistic considering the mutation process, and based on which later different type of mutation-selection (MutSel) model was developed [described below].

In 1994, Yang (Yang, 1994b) developed two approximation approaches for Maximum Likelihood phylogenetic estimation, which allow for varying substitution rates across nucleotide sites. The first, known as the “discrete gamma model,” approximates the gamma distribution by using many rate categories with equal probability for each category. Each category’s mean is employed to depict all of the rates within that category. This method’s performance has been shown to be rather acceptable, with four such categories seeming to be adequate to achieve both an optimum or near-optimal fit by the model to the data, as well as an acceptable approximation to the continuous distribution. The second strategy, dubbed the “fixed-rates model,” divides sites into multiple groups based on the rates anticipated by the star tree. When evaluating alternative tree topologies, sites in various classes are considered to evolve at these constant rates. Analyses of the data sets indicated that this approach might yield good results; however, it seems to have certain aspects with a least-squares pairwise comparison (Yang, 1994b). These models, however, overlook the fact that substitution rates of each amino acid differ distinctly. For instance, as only one transversion is required to convert the phenylalanine codon (UUU) into a leucine codon (UUG) as well as the tryptophan codon (UGG) into a leucine codon (UUG), they consider their substitution rate to be same (Doron-Faigenboim and Pupko, 2007). But in reality, the probability of occurring a former event is approximately 5 times higher than a later event (Doron-Faigenboim and Pupko, 2007).

Considering this lacuna, for the first time, in 2004, Whelan and Goldman developed a complete parametric model that considers several instantaneous substitutions (Whelan and Goldman, 2004). This model estimated substitution rate matrice for single-, double- and triple nucleotide mutation individually utilizing transition to transversion ratio and equilibrated frequency of mutated nucleotides. Later, these three matrices were joined together to estimate the general codon rate matrix. This method is reported to estimate the likelihood of parameters more accurately in comparison to other mechanistic models (Whelan and Goldman, 2004). Double and triple nucleotide substitutions are reported to occur through the mechanistic process, like during repairing DNA break (Sakofsky et al., 2014) or error-prone polymerase activity (Harris and Nielsen, 2014). Although double and triple substitutions rates are predicted to be two to three orders of magnitude lower than single substitutions (Smith et al., 2003; Whelan and Goldman, 2004; Tamuri et al., 2012), the model which included double and triple substitutions were reported to fit better in real data. Later several different models were developed by Doron-Faigenboim and Pupko (Doron-Faigenboim and Pupko, 2007), Kosiol, Holmes and Goldman (Kosiol et al., 2007), De Maio & team (De Maio et al., 2013), Miyazawa (Miyazawa, 2011b), Zoller and Schneider (Zoller and Schneider, 2012), Zaheri, Dib and Salamin (Zaheri et al., 2014), Venkat & team (Venkat et al., 2018) and Jones & team (Jones et al., 2018), which included double and triple substitution between codon.

Later, the model developed *via* Goldman and Yang (Goldman and Yang, 1994) was modified to include various nucleotide models (Pond et al., 2005; Pond and Frost, 2005; Arenas and Posada, 2014), estimate ω variation across sites (Yang, 2007) and branches (Yang, 2007; Dutheil et al., 2012). In the model developed by Pond and Muse, they consider the possibility of site-to-site variation in synonymous and non-synonymous substitution rates in protein-coding DNA sequences and observed that within-gene variability in synonymous substitution rates is common (Pond and Muse, 2005). Another model developed by Mayrose et al. (2007) that uses two hidden Markov models and function on the spatial dimension. First and the second model depicts the dependency between adjacent non-synonymous and synonymous rates rates, respectively. They demonstrate that taking into consideration synonymous rate variability and dependence substantially improves the accuracy of ω estimate, in particular for positively selected sites. In some models, codons were partitioned based on the physicochemical properties of the encoded amino acids (e.g., polarity or charge) (Sainudiin et al., 2005; Wong et al., 2006), codon bias (Yang and Nielsen, 2008) or the effects of GC contents (Misawa, 2011). Models in which codons were partitioned based on the physicochemical properties of the encoded amino acids explicitly parameterized physiochemical constraints due to non-synonymous substitution. The model developed by Goldman & Yang (Goldman and Yang, 1994) and Yang, Nielsen & Hasegawa (Yang et al., 1998) applied mathematical functions for modeling association amongst physiochemical properties and ω parameter. Yang (Yang, 2000) permitted the effect of the physicochemical property to fluctuate among sites. Sainudiin & team (Sainudiin et al., 2005) and Wong & team (Wong et al., 2006) developed two separate models that at first divided non-synonymous substitutions into small groups in accordance with the pre-defined physiochemical property. As the main objective of these two models is to examine the impact of certain physicochemical properties of amino acids on the structure and function of a protein, their parameterization is focused on comparing the property-modifying substitutions rate with the property-conserving substitutions rate. Conant and Stadler (Conant and Stadler, 2009) estimated multiple amino acid properties *via* modeling exchangeability amongst non-synonymous codons as a linear combination of five pre-specified measures of physiochemical properties. This model enabled us to investigate the association between selection pressure and physicochemical properties while avoiding over parameterization of the codon model.

In 2008, Yang and Nielsen (Yang and Nielsen, 2008) developed the FMutSel model in which the amino acid frequencies are determined by the functional requirements of the protein (Rodrigue et al., 2008; Beaulieu et al., 2019). In the FMutSel model, each codon was allocated a fitness parameter. Dissimilarities in fitness parameters amongst two codons are utilized for specifying substitution rates in the Markov matrix *via* altering the rates specified by the standard mutation models (Yang and Nielsen, 2008). The FMutSel/FMutSel0 model combination has only been implemented in PAML4 with the M0 and M3 models so far. Model M0 implies that ω across all branches and sites is constant, whereas Model M3 allows ω to vary between sites (Du et al., 2014). Likewise, in 2010, Rodrigue and the team developed a complex extension of this model in which site-specific amino acid propensity scores are utilized for estimating scaled selection coefficients, which in turn was utilized for identifying substitution rates (Rodrigue et al., 2010). In 2013, De Maio and team (De Maio et al., 2013) reported that when some models were employed to compute ω heterogeneity on data, where both multiple-nonsynonymous rates and double & triple codon modification occur, they yielded high false-positive rates. Recently Venkat & team (Venkat et al., 2018) reported that when branch-site codon models are employed in branch-specific tests to detect positive selection, double modification may cause high false-positive rates. To avoid this problem, recently, Dunn and the team developed a statistically more powerful general-purpose parametric modeling framework for codons (Dunn et al., 2019). By including information about all possible instantaneous codon substitutions, along with instantaneous double and triple nucleotide substitution and multiple non-synonymous rates, both accuracy, as well as statistical power was highly improved (Dunn et al., 2019).

### Empirical codon substitution model

Though empirical codon models are highly useful in understanding protein evolution as well as in phylogenetic applications, only a few models have been developed to date (Kosiol et al., 2007). Substitution rates amongst codons were empirically determined to utilize a large set of protein-coding sequences (Schneider et al., 2005; Kosiol et al., 2007). Unlike mechanistic models, empirical codon substitution cannot distinguish between the substitution rate at the nucleotide level and selective pressure at the protein level (Miyazawa, 2011a). Thus, there is no parameter except codon frequencies for tailoring of each protein family (Miyazawa, 2011a). Delport and team reported that empirical substitution matrices represent average propensities of substitutions across several protein families *via* sacrificing gene-level resolution (Delport et al., 2010).

For the first time in 1990, Schöniger and the team constructed counted codon-codon substitutions matrix on the basis of ∼800 pairwise alignments of 41 actin genes (Schöniger et al., 1990). However, due to a lack of a better electronic facility, this matrix lost its fame in a short interval of time (Cannarozzi and Schneider, 2012). Additionally, as this matrix was developed based on a small number of sequences, it was less reliable. Later in 2005, Schneider and the team (Schneider et al., 2005) developed another empirical codon model utilizing a somewhat similar approach employed by Gonnet and the team (Gonnet et al., 1992) for constructing an amino acid substitution matrix. Since the development of the first amino acid substitution matrix (Dayhoff et al., 1978), it was for the first time Goonet & team (Gonnet et al., 1992) and Jones & team (Jones et al., 1992) developed two individual models based on the sufficiently large amount of sequences and thus are more reliable. In 2007, Kosiol and the team (Kosiol et al., 2007) developed an empirical codon substitution matrix utilizing an extensive database of protein-coding DNA sequences. They reported that the accuracy of the model gets significantly improved by considering instantaneous double and triple substitution. Additionally, the amino acid encoded by each codon, associations amongst codons, and physicochemical properties of amino acids is key factors for driving the process of codon evolution (Kosiol et al., 2007). Empirical codon substitution matrix is reported to outperform mechanistic codon substitution matrix when utilized in likelihood-based phylogenetic analysis (Kosiol et al., 2007). Empirical codon models can also be utilized to detect different lineages sampled in a single phylogenomic dataset (De Maio et al., 2013) rather than depending on a general sequence database for instance Pandit database (Kosiol et al., 2007). In 2014, Bloom developed another novel model that depicts the experimental determination of a parameter-free evolutionary model *via* deep sequencing, mutagenesis, and functional selection (Bloom, 2014). Employing this, Bloom build a model of influenza nucleoprotein evolution that represents the gene phylogeny in a far better way as compared to earlier existing models with nearly hundreds of free parameters. He also emphasized that the data provided by these types of high-throughput experiments can significantly improve the accuracy of both phylogenetic as well as genetic studies.

### Semi-empirical

The semi-empirical codon substitution matrix is often called a mixed empirical codon substitution matrix because it combines empirical substitution rate with mechanistic parameters of codon evolution (Dunn et al., 2019). For the first time, Doron-Faigenboim & Pupko combined existing empirical amino acid substitution matrices with mechanistic parameters (Doron-Faigenboim and Pupko, 2007). They assumed that the substitution rate between non-synonymous substitution amongst codons was equal to the pre-estimated amino-substitution rate, which was obtained by utilizing 189 parameters and a huge amount of amino acid sequences (Doron-Faigenboim and Pupko, 2007). Later Kosiol and team, utilizing 1830 codon substitution parameters and large datasets, developed the first fully empirical codon model and then appended those models with mechanistic parameters for codon evolution (Kosiol et al., 2007). Subsequently, De Maio and team (De Maio et al., 2013), developed another model with almost the same accuracy but was less complex than the model developed by Doron-Faigenboim & Pupko (Doron-Faigenboim and Pupko, 2007). The empirical matrices in those studies denote a wide range of amino acid change propensity (Dunn et al., 2019). Later, Zoller & Schneider (Zoller and Schneider, 2012) and Miyazawa (Miyazawa, 2011a) developed different methods for tailoring information contained in an empirical substitution matrix to a specific dataset, and the benefit of these two approaches is that they can easily distinguish between substitution rate at the nucleotide level and selective pressure at the protein level.

## Applications of the codon substitution model

Codon substitution models are mainly utilized in detecting selective pressure on a protein, codon usage bias, ancestral reconstruction, and phylogenetic reconstruction. All the applications available in recent literature are presented in Table 1.

**TABLE 1:** Applications of codon substitution models.

S. No	Applications	References
1	Identifying heterogeneous selection pressure at amino acid sites	Yang et al. (2000)
2	Identifying molecular adaptation at individual sites along specific lineages	Yang and Nielsen, (2002)
3	Phylogenetic reconstruction	Ren et al. (2005)
4	Codon usage bias	Wu et al. (2007), Yang and Nielsen, (2008), Zhao et al. (2016)
5	Reconstructing ancestral coding sequences	Anisimova and Kosiol, (2009)
6	Molecular dating & functional analysis	Cannarozzi and Schneider, (2012)
7	Evolution of sexual chromosomes, gene families, host-pathogen interactions or regulatory networks	Cannarozzi and Schneider, (2012)
8	Identification and estimation of conservation at synonymous sites	Rubinstein et al. (2012)
9	Detect pathogen evolutionary rate variation	Baele et al. (2016)
10	Identify antibody lineage	Hoehn et al. (2017)
11	Detect evolutionary histories under time-dependent substitution rates	Membrebe et al. (2019)

### Studying selective pressure on a protein

Recent advancements in high throughput sequencing technologies have enabled the generation of a huge amount of sequence data (Gupta et al., 2017; Gupta and Vadde, 2019b; 2020). This enormous amount of sequence data provides an opportunity to detect a direct association between selective pressure and the function of any protein in a more comprehensive way (Anisimova and Kosiol, 2009). Codon models are commonly utilized for identifying candidate genes and their variants under positive selection (Ouyang and Liang, 2007; Parto and Lartillot, 2018; Dunn et al., 2019). Initially, codon models presume that synonymous and non-synonymous substitution rates among sites as well as throughout the phylogenetic history, are constant (Anisimova and Kosiol, 2009). Though the majority of proteins are evolved under purifying selection, the positive selection may affect a few lineages. During this adaptive evolution, only a few protein sites have the capability to increase protein fitness during amino acid substitution (Pupko and Galtier, 2002). Hence, these codon model approaches presuming constant selective pressure over time as well as across sites lack the power to detect genes evolving under positive selection (Anisimova and Kosiol, 2009). Subsequently, several situations of variation in the selective pressure was included with the model developed by Muse & Gaut (Muse and Gaut, 1994) and Goldman & Yang (Goldman and Yang, 1994). These models were later utilised extensively to detect positive selection by likelihood ratio test comparing two nested models. One model (null hypothesis) do not permit positive selection while other model (alternative hypothesis) permit positive positive selection. Positive selection is identified when model permitting sites/lineages under positive selection fits data significantly better than the model restricting the site/lineages under positive selection. Nevertheless, if few parameters become invaluable or because of boundary problems, the asymptomatic null distribution may differ from the standard (Anisimova and Kosiol, 2009).

Additionally, the codon substitution model can be utilized for detecting site-specific positive selection in proteins. Later, this information can be used for testing the biological hypothesis through laboratory experiments (Anisimova and Kosiol, 2009). For instance, in 2005, Swayer and the team reported that a small portion of TRIM5α, an immune defense protein, was recognized to be under positive selection. Later, through functional analysis, they confirmed the significance of the peptide segment in species-specific viral inhibition (Sawyer et al., 2005). The conditional selection model developed by Chen and the team may be utilized particularly for detecting interaction amongst sites during drug resistance (Chen and Lee, 2006). Considering this earlier, we also employed a phylogenetic approach implemented in the PAML’s CODEML modeling tool to identify the kind of selection operating on T2D genes in the Drosophila genus. The data showed that the gene sequences encoding T2D are evolving under purifying selection. However, few membrane protein sites, including those encoded by CG8051, ZnT35C, and kar, are substantially evolving under positive selection. This may be due to adaptive evolution in response to changes in the niche, food, or other environmental conditions (Gupta and Vadde, 2020). Thus, the identification of selective pressure *via* codon substitution models may provide detailed insight into disease progression, pathogenic drug resistance, and epidemic dynamics (Anisimova and Kosiol, 2009).

### Codon usage bias

Gene expression is modulated through transcription (DNA to mRNA) and translation (mRNA to protein) mechanisms (Zhou et al., 2016). Promoter strength & RNA stability are mainly responsible for mRNA concentrations and transcript levels & protein stability is responsible for protein concentrations in any cell (Ikemura, 1985; Sharp et al., 1986). During translation, the information is transmitted as codons. This genetic code is degenerate in nature, i.e., except for tryptophan and methionine, more than one codon (synonymous codons) can encode a single amino acid (Wang et al., 2018). In coding sequences of many organisms, these synonymous codons are utilized at unequal frequencies (Chakraborty et al., 2017). This phenomenon is called codon usage bias. Preferred codons are more frequently utilized in highly expressed genes (Zhou et al., 2016). The degree of codon usage bias differs amongst genes & species and is mainly affected *via* neutral selection, directional mutation, tRNA abundance (Olejniczak and Uhlenbeck, 2006), selection for efficient translation initiation (Zalucki et al., 2007), gene length (Sun et al., 2009), an expression level (Hiraoka et al., 2009), DNA replication initiation site (Huang et al., 2009), *etc.* Codon usage bias can also be utilized in detecting phylogenetic trees amongst species (Wu et al., 2007; Zhao et al., 2016). In 2016, SENCA (site evolution of nucleotides, codons, and amino acids), a codon substitution model, was developed that distinctly describes (a) preferences amongst synonymous codons, (b) amino acids, and (c) nucleotide processes that apply on all sequence sites such as the mutational bias (Pouyet et al., 2016). This model assumes that the vast majority of synonymous substitutions are not neutral and can predict more accurate estimates of selection in comparison to more traditional codon sequence models (Pouyet et al., 2016).

### Ancestral reconstruction

Codon substitution models are also utilized for reconstructing ancestral coding sequences through parsimony and Maximum Likelihood approaches (Anisimova and Kosiol, 2009). These ancestral sequences can further be utilized to detect alterations that have been experienced in every branch of phylogeny and at each individual site of the gene sequence. Several studies have utilized ancestral state information to understand protein evolution and episodic or lineage-specific base composition (Long and Langley, 1993; Akashi, 1996; Eanes et al., 1996; Fitch et al., 1997; Takano-Shimizu, 2001). For instance, the evolution of steroid receptors (Thornton et al., 2003) and ancestral archosaur visual pigment rhodopsin (Chang et al., 2002). Ancestral sequence reconstruction is also employed in studying HIV evolution (Gaschen et al., 2002), protein engineering (Cole and Gaucher, 2011), and understanding variation in DNA turnover because of indels and substitutions amongst eutherian mammalian lineages (Blanchette et al., 2004). Additionally, numerous population genetic tests depend on this ancestral reconstruction to understand the impact of natural selection on the functional classes of mutations or genetic regions (Akashi, 1995; Templeton, 1996; Akashi, 1999; Suzuki and Gojobori, 1999) and also identify coevolving nucleotides/amino acids (Osada and Akashi, 2012; Liao et al., 2013).

### Phylogenetic reconstruction

Codon models reconstruct phylogenetic trees by considering genetic code and the rate of non-synonymous & synonymous base substitutions. In almost every protein-coding gene, the incidence of non-synonymous substitution is less and is mainly involved in early divergence. Synonymous substitutions are higher and are responsible for recent divergence. By considering this information, the codon models may be utilized in reconstructing phylogenetic trees more accurately (Ren et al., 2005). Earlier studies have reported that though nucleotide substitution models are modified to accommodating differences in the evolutionary dynamics at three codon positions (Yang, 1996), the accuracy of this model is lower as compared to codon models. Nevertheless, due to the lack of efficient codon-based tree search methods, tree inference from coding sequence data is generally performed under DNA and AA (amino acid) models. Because of the 61 × 61 matrix, tree generation utilizing codon models is computationally more demanding. To date, no efficient methods have been developed for phylogeny reconstruction utilizing the codon substitution model on a large dataset. For the small dataset, phylogeny can be constructed using CODEML from the PAML package (Yang, 2007). However, Yang has implemented a heuristic algorithm in PAML, which is not the most efficient approach. One possible way to reconstruct an efficient phylogenetic tree is by initially generating numerous phylogenetic trees utilizing both DNA and amino acid substitution models. Later, these trees can be utilized for constructing more accurate trees under efficient Maximum Likelihood (ML) heuristics under codon models (Anisimova and Kosiol, 2009).

Another significant approach in the reconstruction of the phylogenetic tree is by implementing codon models with a Bayesian framework and sampling topological space with an efficient Markov chain Monte Carlo (Anisimova and Kosiol, 2009). By using the Bayesian framework, we can either get similar or even better tree topology in comparison to ML approaches. The main reason for achieving better tree topology *via* the Bayesian framework is that ML approaches search for a single best tree while the Bayesian framework scan cluster of best trees. The benefits of the Bayesian framework can also be explained *via* the matter of probability. The best tree generated *via* ML may ∼90% probability of demonstrating the real information. On the contrary, the Bayesian framework generates hundreds/thousands of near-optimal/optimal having ∼90% probability representing the real information. Hence, the phylogenetic tree generated *via* the Bayesian framework is more realistic than the phylogenetic tree generated *via* ML methods. However, in this Bayesian framework, the rate of substitution will differ for each three codon sites because it considers different data partitions. Thus, codon model usage may serve as an important asset while comparing several candidate trees inferred under either DNA or amino acid models (Anisimova and Kosiol, 2009).

## Limitation and development of next-generation codon model

Though various codon models develop to date provide researchers with a more powerful bioinformatics toolbox, these models’ enormous exchangeability matrices (61 × 61, excluding stop codons) make implementation difficult (Arenas, 2015b). Thus, the development of next-generation development of the codon model with significant attention to model choice as well as the implantation assumptions is highly demanded (Benner, 2012). This can be achieved by using a substantial quantity of data and a considerable amount of computing power. Fortunately, efforts to optimize codon-based algorithms are developing new evolutionary tools for simulating (Fletcher and Yang, 2009; Arenas, 2012) and analyzing (Gil et al., 2013; Zoller et al., 2015) the codons evolution, even though additional research is needed in this area (Arenas, 2015b). In addition to the development of new empirical models, these models may follow two fascinating trends; First, evaluate heterogeneity throughout the sequence and across time since various sites/regions and time periods may evolve differently under distinct models (Arenas, 2015a; Zoller et al., 2015). It is important to remember that these partition methods may be highly realistic, for instance, by using distinct models for coding and non-coding regions. And it is well-known that ω estimations may be influenced by codon models that are based on differences in codon frequency among sites. Thus, there is a need for programs and methods that can determine which codon substitution model works best in a given codon region and time scale (Arenas, 2015b). The second possible trend may be the integration of protein structure data into codon models. Codon models could take into account information about the proteins’ functions and their folding stability (Grahnen et al., 2011; Liberles et al., 2012). But if the protein structure changes over time or if more than one protein structure is required to depict the encoded proteins in the dataset, these implementations would incur high computational costs (Arenas, 2015b).

Considering the limitation above and with the aim to develop more robust codon-substitution models, in 2010, Zoller & Schneider (Zoller and Schneider, 2010) investigated 3,666 codon substitution matrices for detecting the most vital parameters of any codon model. They employed principal component analysis (PCA) to identify the numerous substitution rates that may co-vary across diverse genes. Each individual 3,666 matrices were estimated employing “*XRate”* from a single multiple sequence alignment generated from Mammalian coding sequences. Irrespective of large variance related to parameters computed from very less data, PCA analysis was able to capture a few significant factors. As per PCA analysis, one of the most important parameters in any codon substitution model is the ω value. Amusingly, the substitutions in serine demand two nucleotide alterations and a transitional non-synonymous modification were grouped together with the non-synonymous substitutions. The second most key parameter detected is the ratio amongst substitutions having only one nucleotide dissimilarity and those with two/three dissimilarities. Interestingly, this parameter is not considered in any of the codon-substitution models developed to date. As PCA analysis determines factors that differ maximum in any dataset, there might be an evolutionary use that affects the multi-nucleotide substitutions number that might get stable during coding sequence evolution (Zoller and Schneider, 2010). However, this method was unable to detect other important parameters associated with codon substitution models.

Another study reported that, even though we assume phylogeny on which molecular evolution is modeled is a more appropriate representation of the evolutionary history of any lineage/taxa, but this might not be true in the case of a small dataset or if recombination has been ignored while generating tree topology (Delport et al., 2009). It is possible to include such uncertainty in tree topology *via* Bayesian methods (Yang et al., 2000). For example, MrBayes employed codon substitution models for generating tree topology (Huelsenbeck and Ronquist, 2001). These methods relax the assumption that a specific tree is correct but not the assumption that a correct, though unknown, tree exists. One of the probable solutions for the recombination problem is the introduction of population genetics approximation within the coalescent which co-estimates recombination rate and selective pressure (Wilson and McVean, 2005). Another solution is the identification of recombination breakpoints as well as the prediction of a distinct phylogeny for each individual recombinant. Parameters of these codon models are consecutively calculated in the usual way, except that phylogenies, as well as branch lengths, are partition-specific, while the remaining parameters are shared across all segments (Scheffler et al., 2006). It is also highly advisable to incorporate different synonymous rates in each recombinant because recombination may also lead to differences in synonymous rates (Scheffler et al., 2006). Software, namely, genetic algorithm for recombination detection (GARD), is one of the suitable algorithms for the detection of individual adaptive evolving sites in recombination sequences (Kosakovsky Pond et al., 2006). It is pertinent to note that codon models, particularly those that take rate variation into account, may tolerate modest amounts of recombination (Anisimova et al., 2003; Scheffler et al., 2006). False positive rate estimates may be inflated, however, if recombination rates in such models are very high. Thus, positive selection predictions should be regarded with care for genes with the greatest recombination rates (Davydov et al., 2019).

Earlier several studies have also reported that though codon models developed to date are extensively employed for estimating selective pressure on the gene(s) (MacCallum and Hill, 2006) and scanning genes under positive selection (Li et al., 2010), most of these models generally aimed at investigating the recurrent diversifying selection. Considering this, a few definite models were also developed for investigating the directional selection and were employed on viral data (Kosakovsky Pond et al., 2008; Lacerda et al., 2010). Nevertheless, these directional models are not time-reversible. Recent advancement in sequence technologies enables enormous sequence growth and the development of empirical codon models. Even successful attempts were made to combine empirical estimates along with conventional parameters (Wilson and McVean, 2005).

In addition to phylogeny, codon substitution models can also be employed for studying synonymous codon bias, which may develop because of optimizing for translational kinetics, efficiency, and robustness. Selection against the non-optimal codons often causes a negative correlation amongst synonymous substitution rates and codon bias (Akashi and Eyre-Walker, 1998). Nevertheless, codon bias is generally investigated with different codon adaptation indexes on the basis of single sequences instead of estimating *via* multiple sequence alignment and other parameters of a substitution model. Markov models having fewer states, for instance, codons translated *via* distinct tRNAs, can be employed for studying codon usage as well as asymmetric selective effects (Benner, 2012). On the other hand, mutation and selection may be modeled distinctly for investigating the effects of mutational biases and translational selection (Nielsen and Yang, 2003). Employing such models in 2007, Nielsen and the team investigated the evolution of codon usage over time (Nielsen et al., 2007). In another study, (Yang and Nielsen, 2008) computed optimal codon frequencies as well as mutational bias parameters across multiple species and genes. Further, LRT amongst pairs of nested selection mutation models can be employed for investigating if the codon bias is because of the mutational bias only. This model was further designed to include site specific amino acid profiles, which in turn provide an attractive substitute for fixed as well as random effects models (Rodrigue et al., 2010). Utilizing the Dirichlet process, site-profiles were fitted to the dataset in the Bayesian framework.

**FIGURE 1:**
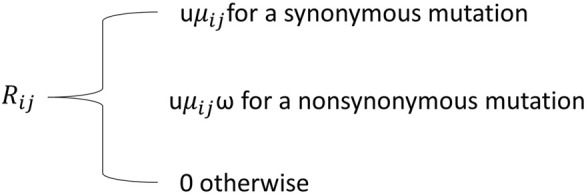
Simple model for substitution rates, where Rij is a nonsynonymous rate, u is a proportionality constant and μij is the rate at which i mutates to j.

One of the underlying presumptions of the codon substitution model is that the rate of codon change is a product of the mutation fixation probability and the mutation rate (Kimura, 1962); this, in turn, forms a significant connection to the population genetic theory. Thus, we may also employ codon substitution models for estimating relationships amongst interspecific and population parameters, e.g., the scaled selection coefficient (Benner, 2012). Given the importance of codon-based models for detecting diversifying positive selection, previous research has focused on two aspects of codon-based models that are important for population genetic interpretations of diversifying positive selection (Thorne et al., 2012). At first, diversified positive selection is a kind of positive selection which is often referred to an allele having a fitness advantage. When alleles’ relative fitnesses are largely consistent across environments, the presence of positive selection is determined by the alleles involved in the substitution rather than the codon position and/or lineage influenced by substitution. On the other hand, codon-based substitution models often seek to identify instances when non-synonymous mutations are beneficial independent of the specific alleles present before and after the mutation. Secondly, diversifying positive selection within codon-based substitution models should be interpreted with care while analysing population genetics. Even though several parameterizations of codon-based models having diversifying positive selection have been developed, they seems have this simple model for substitution rates, as depicted in Figure 1.

Where u is a proportionality constant and μ _ij_ is the rate at which i mutates to j. A population genetic interpretation of a non-synonymous rate R_ij_ would therefore have *ω* proportional to P (Z_ij_), which is the fixation probability approximation by Kimura (Kimura, 1962). One possible way to achieve this is to have all non-synonymous modifications be neutral with respect to selection; however, this would result in ω= 1, which would negate the necessity inclusion for the *ω* parameter. The relative fitness of alleles might also be determined by whether they represent a novel mutation. This would mean that differences in fitness across alleles have nothing to do with the DNA that code for them.

In 2003, Nielsen et al. tried to develop such a model. Interestingly, they allow for variation amongst codon sites. For non-synonymous modifications affecting a specific codon position in a certain lineage, ω was considered to be independent of the decoded amino acids before and after the modification. Since ω was independent of the amino acids involved in the change, Nielsen et al. were able to derive stationary sequence distributions that were independent of the ω value. Since the stationary distribution does not change with codon locations and stationarity can be presumed if the ω value for a branch refers to a small or large population, inferences can be derived more straightforward (Nielsen and Yang, 2003). It is pertinent to note that inference of stationary distribution was also possible before as in (Nielsen and Yang, 2003), however not much studies have been done. Earlier, Halpern and Bruno (Halpern and Bruno, 1998) also developed the MutSel model to unmask the mechanistic, population-genetic explanation of evolution. In this method, a nucleotide mutation model that is the same for all sites is combined with fixation probability calculated from site-specific vectors of fitness coefficients under the assumption of a Wright-Fisher population with mutation and selection (Jones et al., 2017). This framework offers a systematic approach to generating realistic sequence alignments that are capable for detecting positive selection by directly relating ω to fitness differences across amino acids. By forcing changes in fitness coefficients at predetermined sites and branches, extensions of the MutSel model (dos Reis, 2015) can also capture episodic positive selection. Irrespective of all these advancements, implementation of population genetic theory in the codon models is still in the infancy stage (Benner, 2012). One important challenge is how to differentiate between episodic changes in fitness landscapes and shifting balance in the model. Positive selection *via* shifting balance is an autonomous, unpredictable, and site-specific mechanism. So, the key question is, how often is shifting balance in real-world data? (Thorne et al., 2012; Jones et al., 2017). Another difficulty is posed by the fact that mutation-selection equilibrium may be disrupted by a wide variety of population genetic processes and how to include all these parameters in the model (Thorne et al., 2012).

There is also still scope for Monte Carlo approach development. Specifically, to date, “data-augmentation-based” methods have received very little attention in terms of codon substitution model development. This “data-augmentation-based” is though short-lived but has computational benefits (de Koning et al., 2010). For instance, thermodynamic integration is computationally expensive and, hence, not much used in molecular evolutionary or Bayesian phylogenetic applications. This is why the harmonic mean estimator (HME), which has an infinite variance and produces less reliable results (Lartillot and Philippe, 2006), is still widely used. Advancement in this direction, nevertheless, is also in full swing. For example, in 2011, Xie and the team (Xie et al., 2011) developed a more robust method, namely, the “stepping-stone method”, on the basis of similar concepts, though employing a discrete path in preference to a continuous one. In the near future, there is also scope for combining “thermodynamic-based” methods with “data-augmentation based” approaches. The “stepping-stone” approaches, along with other recently developed computational methods, may also contribute significantly to developing Bayes factor, thereby providing a wide-range evaluation of the performance of numerous different codon substitution modeling methods.

In 2010, Du and the team proposed new codon-based ancestral reconstruction approaches that permit to examine changes in codon usage bias in rhodopsin, which in turn might be responsible for shifts in the visual ecology within the early mammals (Du, 2010). Using the same approach, they observed an evolutionary trend towards enhanced GC-ending codons at three early mammalians, i.e., therian, placental and mammalian lineages of rhodopsin. However, they also proposed that there is still scope for incorporating a Bayesian distribution of different ancestral states while estimating the Akashi ratio for calculating deviations from equilibrium codon usage, as well as simulations for accessing the significance of the deviations detected for rhodopsin (Du, 2010).

In one study, authors proposed that augmenting codon model application along with information obtained from other approaches, for instance, population genetics, coalescence, and HMMs may enable us to understand the evolution of the complex system in a more comprehensive way. For instance, in 2010, Gilbert and Parker proposed a codon substitution model that can be used extensively to study the origin of fungal diseases; specifically, that are associated with crops (Gilbert and Parker, 2010). When any fungi are exposed to a novel environment in a new host, they evolve very fast. Using these new codon models, we can predict pesticide targets on the basis of the nature of selection acting on crucial genes. These models can also be employed for investigating the novel function of regulatory genes as well as networks and important pathways associated with pathogenesis (Benner, 2012). Recently, several other studies have also proposed a new hypothesis in the context of intracellular pathogens (Casadevall, 2008). As per that hypothesis, fungi become intracellular pathogens *via* dual-use traits evolution. For instance, genes originally associated with escaping amoeba predation consequently became advantageous and helped in invading animal or plant cells (e.g. adhesins, toxins, efflux pumps, and injectors, among others) (Benner, 2012). Codon models can also be employed for tracing selective pressure acting on dual traits under diverse circumstances (Benner, 2012).

Some researchers have also proposed that functional divergence of proteins subsequently after some events, for instance, gene duplication, may also result in complex sequence evolution, which is poorly described *via* presently available “branch-site” codon models (Anisimova and Liberles, 2007; Benner, 2012). On the contrary, recently developed clade models, Clade model C (CmC) & Clade model D (CmD) (present in the CODEML utility of the PAML software package (Yang, 2007), are a collection of flexible “codon-substitution” models comprised of both “among-lineage” as well as “among-site” variation in selective pressure, which in turn can be an effective tool for investigating signatures of functional divergence amongst clades (Bielawski and Yang, 2004). To date, the clade models have been utilized for studying functional divergence in numerous gene families, e.g., β-globins (Aguileta et al., 2004) and vertebrate Troponin C (Bielawski and Yang, 2004).

When augmented with EB site assignment methods, these clade models may also provide an opportunity to unmask the molecular bases of functional diversification, as well as help in understanding biochemical analyses of homologous yet functionally divergent proteins (Benner, 2012). However, these clade models are still in the infancy phase and further research is required to establish actual power as well as accuracy while dealing with complex forms of divergence among clades (Benner, 2012). Nevertheless, one most important limitations of the present clade models is the absence of incorporation of “among-site rate variation” within *ω*. At present, both CmC and CmD presume only one site class for which *ω* either decreases or increases (but not both). But in reality, a large number of complex divergence scenarios are possible. For example, a few sites present within the divergent clade may switch to neutral from purifying class, while others may switch in the opposite direction (Benner, 2012). If such a scenario exists, novel approaches for detecting might be necessary as like the ‘switching’ codon models developed *via* Guindon and the team (Guindon et al., 2004).

Thus, by augmenting new parameters to existing codon substitution models or by designing novel algorithms, we can develop more robust and less computationally demanding codon substitution models for more accurate phylogeny as well as understanding the evolutionary history of any sequences or organisms.

## Conclusion

wing to the presence of the huge amount of genomic sequences due to recent advancements in technology, it is easy to understand the evolutionary history of any sequences or organisms in a far better way. Phylogenetic analysis utilizing nucleotide/amino acid/codon substitution models are the most powerful tool for unraveling the evolutionary history of genomic sequences/organisms. However, in comparison with nucleotide and amino acid models, the codon substitution model is more powerful. These models have been utilized extensively to detect selective pressure on a protein, codon usage bias, ancestral reconstruction and phylogenetic reconstruction. However, most of the codon substitution models are still in their infancy stage and deserve further attention. On the downside, the presence of a large variety of models and each considering different biological factors, enhances the margin for misinterpretation. The biological meaning of certain parameters may differ amongst models and thus, model selection procedures also deserve greater attention. Additionally, due to more computational demanding, in comparison to nucleotide and amino acid substitution matrices, only a few studies have employed the codon substitution model to understand the heterogeneity of the evolutionary process in genome-scale analyses. Thus, there is still scope for developing more robust and less computationally demanding codon models. Authors believe that a more robust codon substitution model can be developed considering parameters like the size and structure of the population across time and uncertainty in the ancestral state during estimation. Additionally, results obtained from these models, when combined with other multidisciplinary approaches, like epidemiology, physiology, and molecular biology, are most likely to detect selective pressure on a protein, codon usage bias, ancestral reconstruction and phylogenetic reconstruction in a more comprehensive way. Thus, it seems clear that, in the near future, research on substitution models requires the design and development of more sophisticated as well as realistic substitution models. For instance, the development of codon models with more relaxing assumptions like temporal heterogeneity in both mutational as well as selective processes. Additional effort is also being required to evaluate, compare and apply these newly developed models with real large datasets. As the codon substitution model enables to detect selection regime under which any gene or gene region is evolving, codon usage bias in any organisms or tissue-specific region and phylogenetic relationship between different lineages more accurately than nucleotide and amino acid substitution models, in the near future, these codon models can be utilized in the field of conservation, breeding and medicine.
